# Obstinate Vomiting in Pregnancy

**Published:** 1882-02

**Authors:** 


### Obstinate Vomiting in Pregnancy.

At a meeting of the Obstetrical Society of Boston, Dr. Fifield said that for several years he had prided himself on being able to subdue this symptom either by the administration of the bromide of potassium or by the injection of chloral hydrate, one half drachm, into the rectum. These had never failed him till about a week before the meeting, when the case of a woman, three or four weeks pregnant, fell into his hands. She had been vomiting for several weeks before he saw her, and then was rejecting everything. Dr. Fifield first tried the bromide, then the chloral, with the simplest diet, then ingluvin, ten grains at five or six A. M., fifteen at nine o'clock, iced milk bubbled with water from the siphon, and a slice of bread; the second morning the same plan, the third day three grains three times, as directed by an English gynæcologist. Meanwhile, so far from amending the patient got into an alarming state, and began to vomit blood. On the morning of the day before the meeting Dr. Fifield applied Sims's speculum, drew down the cervix, a little excoriated, and covered it thoroughly with nitrate of silver. He then ordered bromide of potassium, ten grains, every two hours. The next day she was well.

Dr. Blake remarked that the most obstinate cases of vomiting in pregnancy had been associated with abrasions of the cervix.

Dr. Benjamin Cushing said he had seen in his own practice of twenty-eight years two cases only which threatened a fatal termination. In the first case the patient seemed in danger of starvation, she also had convulsions. Dr. Cushing gave as the last, and apparently effectual, remedy fifteen drops of Smith and Melvin's elixir of opium, twenty minutes before each mill. The vomiting ceased, and the patient went her full time. In the second case all remedies taken internally failed. The os uteri was dilated with sponge-tent, miscarriage followed, and the patient recovered.

Another case was mentioned where long, obstinate vomiting, not due to pregnancy, was followed by relief on using suppositories of sulphate of morphine. One quarter of a grain was given at bed-time for a few nights, and then the quantity gradually diminished. The morphine was used for about three weeks.

Dr. W. Symington Brown remarked that he had seen two fatal cases of the vomiting of pregnancy, one of which was reported in the *Journal* of the Gynæcological Society of Boston, Vol. II, p. 208. The other occurred in the practice of the late Dr. Wm. F. Stevens. Abortion was induced by means of a sponge-tent, but the patient died. The emesis was somewhat relieved by the operation.

Dr. Abbot had seen one fatal case, in which the most relief had been found from large doses of morphia and dilatation of the cervix. He called attention to the fact that abortion is not allowed the Catholic patient as a means of relief. The patient referred to belonged to that church.

Dr. Lyman referred to a severe case which was treated by Copeman's method; abortion accidentally ensued, and the patient got well.—*Boston Medical and Surgical Journal.*

